# Quantitative Proteomics Identifies DNA Repair as a Novel Biological Function for Hepatocyte Nuclear Factor 4α in Colorectal Cancer Cells

**DOI:** 10.3390/cancers11050626

**Published:** 2019-05-05

**Authors:** Jean-Philippe Babeu, Samuel D. Wilson, Élie Lambert, Dominique Lévesque, François-Michel Boisvert, François Boudreau

**Affiliations:** Department of Anatomy and Cell Biology, Université de Sherbrooke, 3201 Rue Jean-Mignault, Sherbrooke, QC J1E 4K8, Canada; Jean-Philippe.Babeu@USherbrooke.ca (J.-P.B.); Samuel.Wilson@USherbrooke.ca (S.D.W.); Elie.Lambert@USherbrooke.ca (É.L.); Dominique.Levesque@USherbrooke.ca (D.L.)

**Keywords:** HNF4α, SILAC, BioID, proteomics, DNA repair, etoposide

## Abstract

Hepatocyte nuclear factor 4α (HNF4α) is a transcription factor that acts as a master regulator of genes for several endoderm-derived tissues, including the intestine, in which it plays a central role during development and tumorigenesis. To better define the mechanisms by which HNF4α can influence these processes, we identified proteins interacting with HNF4α using stable isotope labelling with amino acids in cell culture (SILAC)-based quantitative proteomics with either immunoprecipitation of green fluorescent protein (GFP) or with proximity-dependent purification by the biotin ligase BirA (BioID), both fused to HNF4α. Surprisingly, these analyses identified a significant enrichment of proteins characterized with a role in DNA repair, a so far unidentified biological feature of this transcription factor. Several of these proteins including PARP1, RAD50, and DNA-PKcs were confirmed to interact with HNF4α in colorectal cancer cell lines. Following DNA damage, HNF4α was able to increase cell viability in colorectal cancer cells. Overall, these observations identify a potential role for this transcription factor during the DNA damage response.

## 1. Introduction

The transcription factor hepatocyte nuclear factor 4α (HNF4α) regulates the development and differentiation of epithelia in digestive and accessory digestive organs [1,2,3]. Within the digestive system, HNF4α is expressed in the epithelia of the liver, pancreas, stomach, small intestine, and colon [4]. Originally identified in the liver, HNF4α is a member of the superfamily of nuclear receptors, NR2A1 [5]. HNF4α controls the expression of genes involved in epithelial differentiation that are necessary for liver and pancreatic tissue [6] and important for the intestinal tissue [1,7,8]. HNF4α had originally been considered a tumour suppressor protein for its roles in differentiation and interplay with the Wnt/β-catenin pathway [7,9,10]. Some later research suggested HNF4α to act as an oncoprotein in the intestine [11,12]. This apparent paradox could be explained by the existence of two isoform classes produced by two different promoters, namely P1 promoter-driven and P2 promoter-driven HNF4α isoforms [13]. It has recently been suggested that P1-HNF4α exerts a differentiative effect on intestinal epithelial cells while P2-HNF4α exerts a proliferative effect on these cells [14,15].

An aberrant expression of the isoform classes of HNF4α seems to be implicated in oncogenesis. The HNF4α expression is altered in cancers of all the tissues where it is expressed. More specifically, an increase in the P2-HNF4α expression coincides with a cancerous state [4]. The P2-HNF4α expression is increased in colorectal cancer while the P1-HNF4α expression is often lost in affected patients. Exacerbation of intestinal tumorigenesis was observed in exon swap mice expressing only P2-HNF4α [16].

For colorectal cancer, and most other cancers, an abnormal DNA repair response underlies the initiation and progression of oncogenesis. Colorectal cancer features genomic instability that can typically be categorized into three subtypes: Chromosomal instability (CIN), microsatellite instability (MSI), and CpG-island methylator phenotype (CIMP) [17]. These genomic instabilities usually result from faulty genetic material that give rise to deficient DNA damage response in cancerous cells. Transcription factors can affect DNA repair indirectly by regulating the expression of genes encoding components of different DNA repair pathways [18]. Probably the best-characterized example is the initiation of the transactivation activity of p53 transactivation activity through the phosphorylation of its serine 20 by CHK2 and CHK1 and of its serine 15 by ATM/ATR [19,20]. The latter are important DNA damage sensors and can also regulate MDM2 degradation by ubiquitination, resulting in the stabilization of p53 [21]. These mechanisms converge to increase the transactivation activity of p53 and therefore the expression of several genes coding for proteins involved in cell cycle regulation, DNA repair, and apoptosis [22]. However, an increasing number of studies have now demonstrated that several transcription factors can also regulate DNA repair directly through interaction with DNA repair proteins, and through recruitment to DNA lesions [23]. ATF2, a transcription factor from the AP1 family of stress response effectors, regulates the expression of several genes functioning in DNA repair [24]. The proposed mechanisms for these transcription factors’ involvement in DNA repair is still unclear, but their DNA binding seems to be required for effective DNA repair. 

In this study, we sought to investigate the roles played by the P2-HNF4α isoform class in colorectal cancer by identifying its interactome. Using SILAC-based quantitative proteomics approaches [25], we identified proteins associated with the DNA damage response unexpectedly as interactors of P2-HNF4α. These results, combined with the increase of CRC cells viability to DNA damage in the presence of HNF4α, support a functional role for this transcription factor during the DNA damage response.

## 2. Results

### 2.1. Generation of Cells Stably Expressing A Transcriptionally Functional P2-HNF4α Fusion Protein Construct

The gene *HNF4A* can produce two classes of isoforms from two unique promoters, designated P1 and P2 [13]. Recent biological data have supported differential roles for these two classes of proteins in the colon with P1-HNF4α being functionally involved in suppressing the colon tumorigenesis [14,15,16] and P2-HNF4α being associated with cell proliferation and human colon cancer [4,14,16,26]. The nature of the specific transcriptional targets for HNF4 α has been studied in multiple tissue contexts, including the intestine [14,15,27,28]. To identify novel putative functions for HNF4α, we decided to explore its protein interactome.

**Figure 1:**
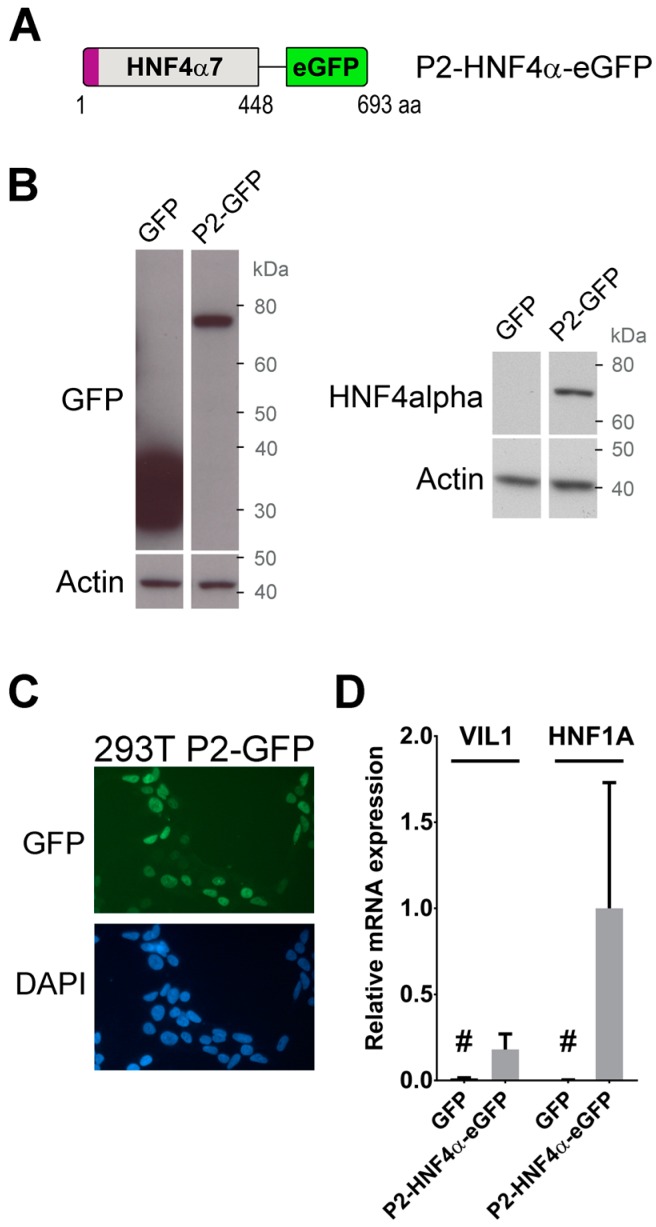
The P2-GFP construct displays transcriptional functions associated with hepatocyte nuclear factor 4α (HNF4α) *in cellulo*. (**A**) Structure of the bait used for the GFP-Trap assay. The sequence of eGFP was fused at the C-terminal end of HNF4α7 isoforms and separated by a linker sequence of six amino acids; (**B**) expression of the P2-GFP construct was tested by infecting HEK293T cells with lentivirus expressing P2-GFP, or the GFP tag alone. Total protein extracts were analyzed by western blot using an antibody against GFP (left panel) or against HNF4α (right panel); (**C**) nuclear localization of P2-GFP was determined by fluorescence microscopy on HEK293T cells stably expressing the construct after lentiviral infection. Nuclei were counterstained using DAPI; (**D**) total RNA was extracted from P2-GFP HEK293T cells or control GFP cells and the expression of *VIL1* and *HNF1A* gene transcripts levels measured by qPCR. Expression was normalized to the *MRPL19* housekeeping gene. P2-GFP are *n* = 3, GFP control are *n* = 2. # = background noise since these genes are not expressed in HEK293T cells.

We focused on identifying protein partners of P2-HNF4α based on its potential functional role as an oncogene during the colon tumorigenesis growth. A gene cassette for HNF4α7 (NCBI; denoted as α8 by UniProt) was synthesized to which an eGFP gene cassette was added on the C-terminus (Figure 1A) (P2-GFP) and inserted into the pLenti6/V5 vector for expression by the lentiviral infection. The HEK293T cell line was chosen as a model because of its relative ease for overexpression of recombinant protein constructs and because the cell line does not endogenously express HNF4α despite it originating from the human kidney epithelium. The expression of P2-GFP was first confirmed in the transduced population of HEK293T cells by immunoblotting against GFP and HNF4α. In cells expressing P2-GFP, a single band appeared around the expected size of the fusion protein (77 kDa) (Figure 1B). Immunofluorescence against GFP was next performed to ensure that the recombinant protein was able to localize in the nucleus of HEK293T cells. As expected for HNF4α, a fluorescent signal was strictly detected in the nucleus of HEK293T-transduced cells when compared to nuclear specific DAPI staining (Figure 1C). *VIL1* and *HNF1A* gene transcript expression was next assessed by qPCR in the HEK293T-transduced cells to confirm that the inclusion of the eGFP tag did not interfere with the activation of target gene expression [1,8]. The expression of both *VIL1* and *HNF1A* gene transcripts was specifically induced only when P2-GFP was expressed in HEK293T cells as compared to controls with only eGFP (Figure 1D). Similarly, the P2-GFP expression led to an induction of *VIL1*, *CYP17A1*, *PC*, *CDKN1A* and *CDH1* gene transcripts when forced in HCT116 cells (Figure S1) [29]. These observations confirmed that the recombinant P2-GFP mimicked endogenous transcriptional functions associated with HNF4α and could therefore be used as a functional model for studying the factor’s protein interactors.

### 2.2. Novel P2-HNF4α Protein Interactomes Identified by Quantitative Proteomics in HEK293T Cells

**Figure 2:**
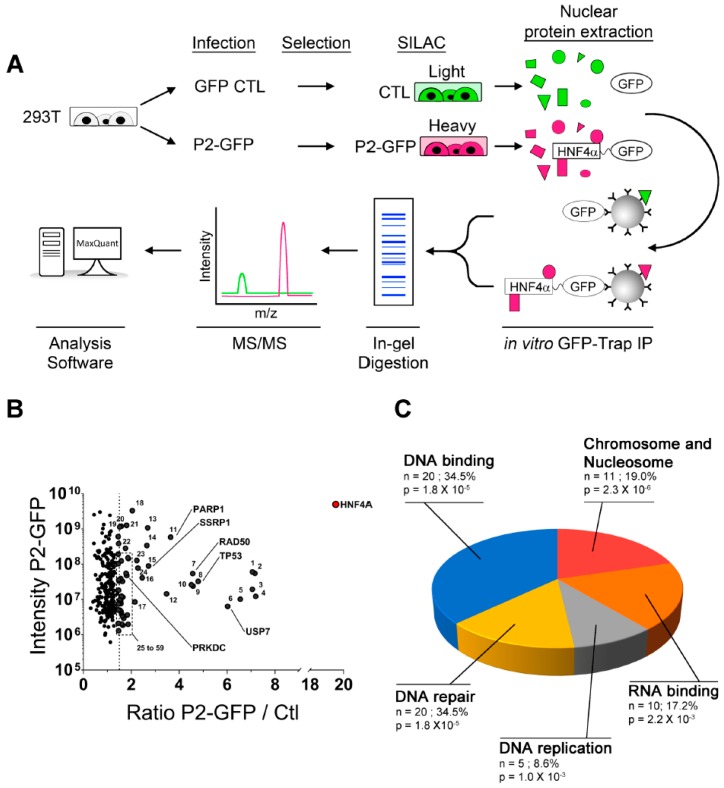
GFP-Trap analysis of P2-GFP in HEK293T identifies DNA damage proteins as partners. (**A**) GFP-Trap experimental strategy for the identification of new interacting partners of P2-GFP. HEK293T cells were infected with lentivirus containing a vector expressing the P2-GFP construct or an empty GFP vector as control. After seven days of selection, the proteome of each cell line was labelled using light (CTL) or heavy (P2-GFP) isotopes of lysine and arginine using the SILAC technique. To identify proteins interacting with the P2-GFP construct, nuclear proteins were isolated from each cell line and incubated for one hour with agarose beads coated with anti-GFP antibodies. Pulldown proteins in each condition were mixed together, separated by SDS-PAGE and cut down to peptides by an in-gel digestion with trypsin. Peptides were analyzed by MS/MS and proteins were identified according to the molecular signature from the isotope labelling using the MaxQuant software; (**B**) distribution of the interactors enriched in the P2-GFP pulldown assay. The enrichment ratio for each protein found in the P2-GFP pulldown compared to the CTL condition was plotted against their MS intensity in the P2-GFP condition. Using an enrichment cutoff of 1.5 fold (dotted line), 59 proteins were identified among which many are implicated in the DNA damage response. The graphic illustrates the combined results of two independent GFP-TRAP assays; (**C**) biological functions associated with the interactors identified by GFP-TRAP. The identified 59 interactors were analyzed by DAVID to characterize their biological functions. *n* = number of proteins identified that are associated to that function, % = percentage of the protein pulldown with P2-GFP and that are associated with that function, *p* = Benjamini corrected *p*-value.

**Figure 3:**
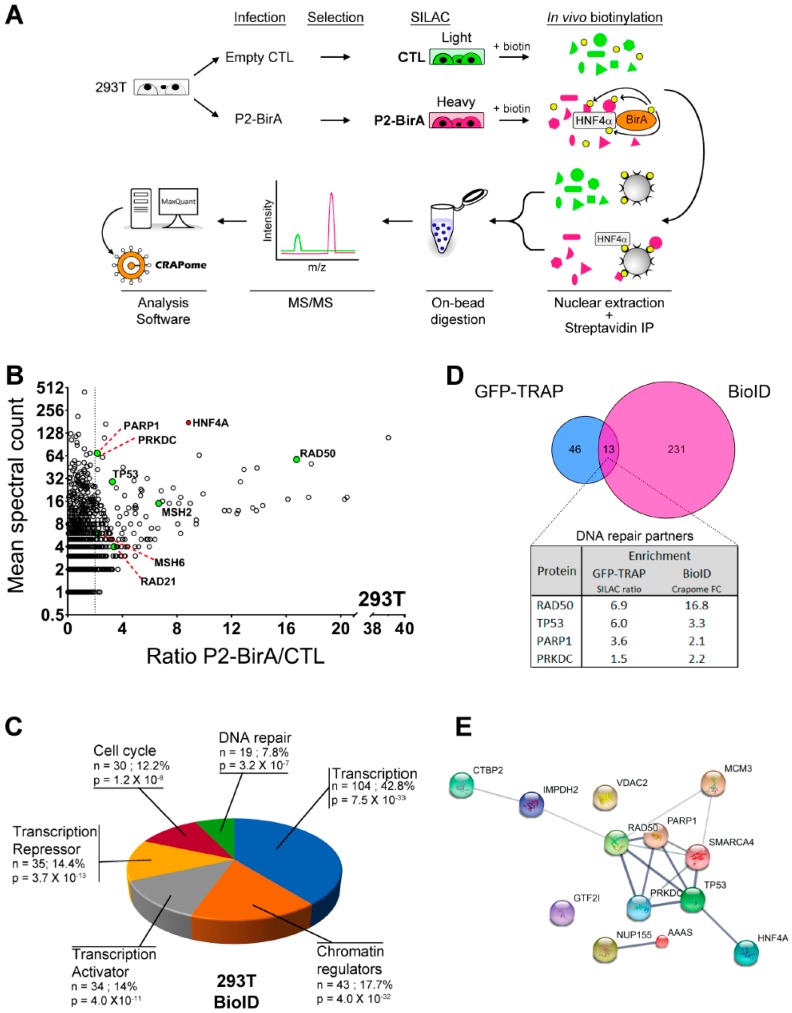
BioID analysis confirms interaction of P2-BirA with DNA damage proteins in HEK293T cells. (**A**) Experimental design of the BioID technique used to label in vivo interacting partners of P2-BirA. HEK293T cells were infected using lentivirus expressing a construct of HNF4α tagged in C-terminal with the biotin ligase enzyme *BirA* (P2-BirA) or a control empty vector. After selection, the whole cell proteome was labelled by SILAC using light molecular weight isotopes for control and heavy molecular weight isotopes for P2-BirA cells. Cells were then incubated for 24 h in the presence of 50 µM of biotin allowing endogenous biotinylation of proteins surrounding the P2-BirA recombinant protein. Nuclear proteins were then extracted, and biotinylated proteins were purified using the affinity capture by streptavidin-coupled beads. The beads from the two conditions were then combined to fragment proteins in smaller peptides by on-bead digestion using trypsin. Peptides were analyzed by MS/MS and proteins were identified according to the molecular signature from the isotope labelling using the MaxQuant software and enrichment above background calculated by the CRAPome database; (**B**) distribution of the proteins biotinylated by P2-BirA in the HEK293T cells. The enrichment ratio of each protein in the P2-BirA condition was plotted against its mean spectral count. The proteins that are implicated in the DNA damage response have been highlighted; (**C**) comparison of the partners of P2-HNF4α identified by GFP-Trap or BioID. Among the 13 common targets identified, the DNA repair proteins TP53, RAD50, PARP1 and PRKDC (DNA-PKcs) were found; (**D**) biological function analyses by the software DAVID of the proteins biotinylated by P2-BirA in HEK293T cells. *n* = number of proteins identified that are associated to that function, % = percentage of the protein pulldowns with P2-GFP that are associated with that function, *p* = Benjamini corrected *p*-value; (**E**) interaction relationship between the 13 common targets identified by GFP-Trap and BioID based on the STRING database. Line thickness indicates the strength of the predicted relationship between the proteins.

An in vitro affinity capture assay coupled to SILAC quantification of interacting proteins was next designed in HEK293T cells with the use of the HNF4α7-eGFP recombinant protein as bait (heavy) and eGFP alone used as a control (light) to subtract non-specific interactions (Figure 2A). P2-GFP and GFP were both immunoprecipitated with GFP-Trap agarose beads and were subsequently identified by mass spectrometry (Figure 2A) through two biological replicates, each with two technical replicates. This analysis identified 59 proteins enriched more than 1.5 times in P2-GFP pulldowns when compared to GFP controls (Figure 2B and Table S1). The gene ontology analysis of the enriched targets was performed with DAVID [30,31] and identified the most significant biological processes as being related to Chromosome and Nucleosome maintenance (*p* = 2.3 × 10^−6^), DNA binding (*p* = 1.8 × 10^−5^) and DNA repair (*p* = 1.8 × 10^−5^) (Figure 2C). Interestingly, some of these protein partners, such as PARP1, RAD50 and PRKDC (DNA-PKcs) (Figure 2B), have not been functionally ascribed to the gene transcription but to enzymatic functions within DNA repair. To further validate the possible involvement of P2-HNF4α in recruiting enzymatic DNA repair complexes, we took advantage of a complementary approach using the proximity purification of proteins coupled to mass spectrometry (Figure 3A). P2-HNF4α was fused to a BirA* tag [32] and, as observed for the P2-GFP construct, the fusion protein was able to induce the expression of *VIL1* and *HNF1A* gene transcripts in HEK293T cells (Figure S2). Endogenous biotinylated proteins were purified with streptavidin pulldowns and the quantitative analysis of mass spectrometry identified 244 proteins enriched more than two times when compared to controls (Figure 3B and Supplementary Table S2). The gene ontology analysis for biological process enrichment found Transcription (*p* = 7.5 × 10^−33^), Chromatin regulators (*p* = 4 × 10^−32^), Cell cycle (*p* = 1.2 × 10^−8^) and DNA repair (*p* = 3.2 × 10^−7^) as the most significant biological functions associated with the protein pulldowns (Figure 3C). A comparison of the protein pulldowns from both GFP-Trap and BioID approaches identified 13 common targets for which four of them were directly linked to the DNA damage response (Figure 3D). Analysis of known interactions and connections between the proteins identified as interacting with HNF4α using both approaches identified a node of proteins involved in DNA repair, suggesting a possible link with this protein complex (Figure 3E). 

### 2.3. HNF4α Recruits DNA Damage Response Protein Partners in Colorectal Cancer Cells

**Figure 4:**
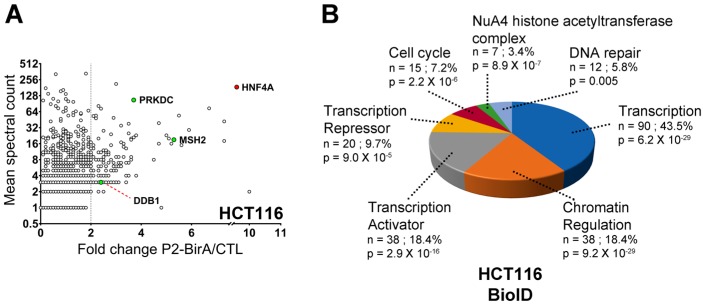
BioID analysis of P2-BirA in the colorectal cancer cell line HCT116 reveals interaction with DNA repairs proteins. (**A**) Distribution of the proteins biotinylated by P2-BirA in the HCT116 cells. The enrichment ratio of each protein in the P2-BirA condition was plotted against its mean spectral count. The proteins that are implicated in the DNA damage response have been highlighted; (**B**) biological function analysis by the software DAVID of the proteins biotinylated by P2-BirA in HCT116 cells. *n* = number of proteins identified that are associated to that function, % = percentage of the protein pulldowns with P2-GFP that are associated with that function, *p* = Benjamini corrected *p*-value.

**Figure 5:**
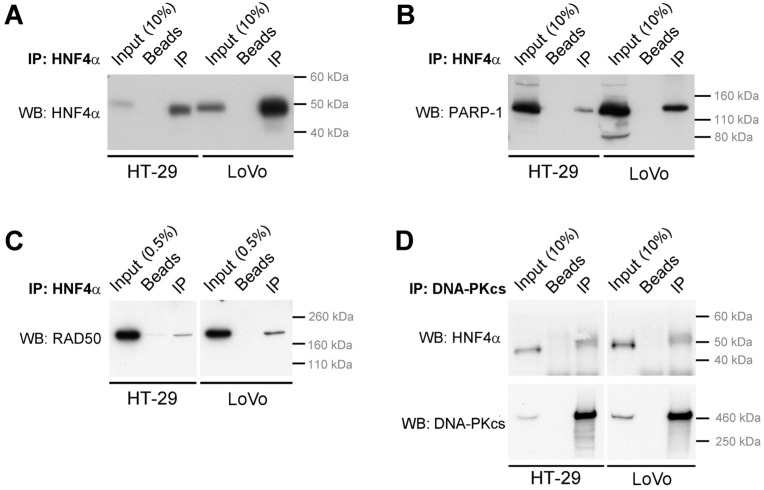
DNA repair proteins co-immunoprecipitate with endogenous HNF4α in colorectal cancer cells. Total protein extracts were isolated from HT-29 and LoVo colorectal cancer cell lines, and HNF4α (**A**–**C**) or DNA-PKcs (**D**) were immunoprecipitated using specific antibodies. Following immunoprecipitation, the specific enrichment of HNF4α, PARP-1, RAD50, or DNA-PKcs in the immunoprecipitate was verified by Western blot. In both cell lines, PARP-1, RAD50, DNA-PKcs were detected as proteins co-immunoprecipitated along with HNF4α. Incubation of protein extracts with beads alone served as control for the immunoprecipitation.

To further explore the relevance of the capacity of HNF4α to recruit the DNA damage response protein partners in colorectal cancer, HCT116 cells that are devoid of the HNF4α expression [11,33] were used to stably express the P2-BirA recombinant protein and to perform a BioID analysis similarly to what was done in the HEK293T cells. HCT116 endogenous biotinylated proteins were pulldown and the quantitative analysis of mass spectrometry identified 208 proteins enriched more than two times when compared to controls (Figure 4A and Table S3). Again, DNA repair was identified as one of the significant biological functions associated with pulldown biotinylated proteins based on the gene ontology analysis for biological process enrichment (Figure 4B). We then sought to determine whether the DNA damage response proteins could interact with the endogenous HNF4α. HT-29 and LoVo colorectal cancer cell lines were chosen since they both expressed the endogenous HNF4α [11]. The endogenous HNF4α was immunoprecipitated from both HT-29 and LoVo cell extracts (Figure 5A) and the detection of the DNA damage response protein partners identified (Figure 2, Figure 3 and Figure 4) was performed by the co-immunoprecipitation analyses. PARP-1, a protein involved in the response to single and double strand DNA breaks and important to many cancers [34], was confirmed for interaction after pulldown of the endogenous HNF4α (Figure 5A) from both HT-29 and LoVo cells (Figure 5B). A similar observation was made for RAD50 (Figure 5C), a protein crucial for MRN complex enzymatic activity during the DNA double-strand break repair [35]. Immunoprecipitation of DNA-PKcs, the catalytic subunit of a nuclear DNA-dependent kinase required for NHEJ [36], was also able to pulldown the endogenous HNF4α from both HT-29 and LoVo cells (Figure 5D).

**Figure 6:**
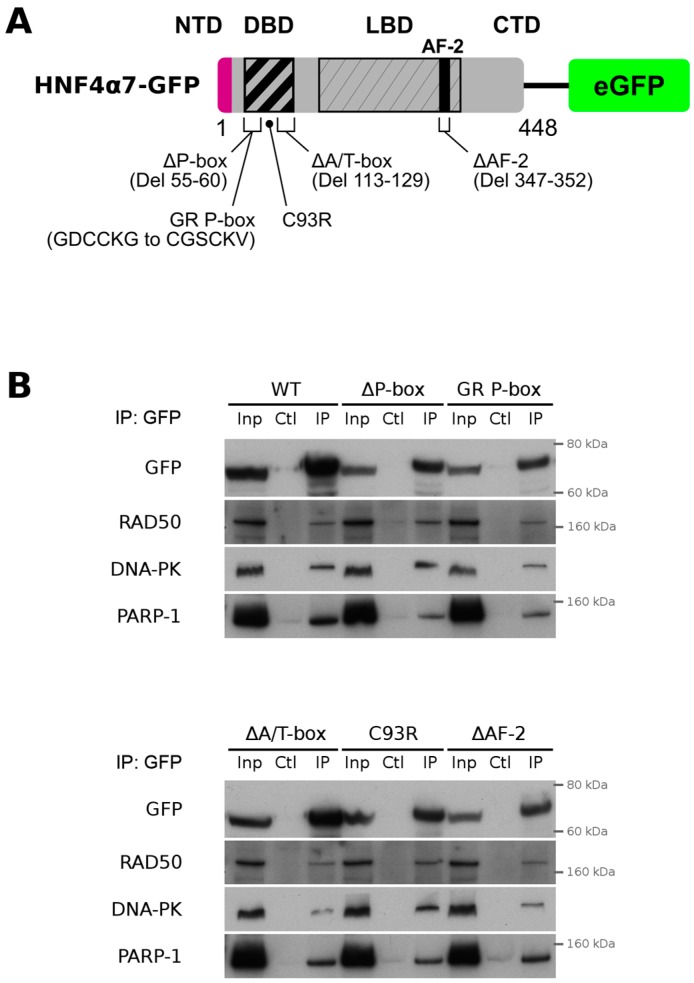
P2-GFP interaction with DNA repair proteins is independent of the DNA binding or AF-2 domains. (**A**) Representation of the P2-GFP mutant constructs used in co-immunoprecipitation assays. The DNA binding domain (DBD) of the wild-type P2-GFP was mutated to inactivate its activity by deleting a portion of the first zinc finger (ΔP-box), by creating a point mutation of a cysteine in the second zinc finger (C93R) or by deleting the A-box and T-box sequences (ΔA/T-box). The DBD activity was also altered by substituting the P-box sequence of HNF4α with the one from the glucocorticoid receptor (GR P-box). The cofactor interacting domain AF-2 was also deleted (ΔAF-2) to reduce the cofactors binding capacity; (**B**) HCT116 cells were transfected with the wild-type or mutant P2-GFP constructs. Total protein was isolated 48 h following the transfection and P2-GFP recombinant proteins were immunoprecipitated using GFP-Trap beads. Following immunoprecipitation, the specific enrichment of P2-GFP constructs, RAD50, DNA-PKcs or PARP-1 was determined by Western blot. Inp = input, Ctl = immunoprecipitated samples from control sepharose beads, IP = immunoprecipitated samples from GFP-Trap beads.

To further test whether the DNA binding domain (DBD) or the AF-2 transactivation domain of P2-GFP was necessary for its interaction with DNA repair proteins, various mutant constructs within the DBD and the AF-2 region were generated (Figure 6A). None of the P2-GFP mutants tested failed to interact with the RAD50, DNA-PK or PARP-1 proteins when exogenously expressed in the HCT116 cell line that do not endogenously express HNF4α [33] (Figure 6B). These observations suggested that the transcription relevant domains of HNF4α were not essential to physically interact with DNA repair proteins.

### 2.4. P2-GFP Decreases Sensitivity to Etoposide in HCT116 Cells

**Figure 7:**
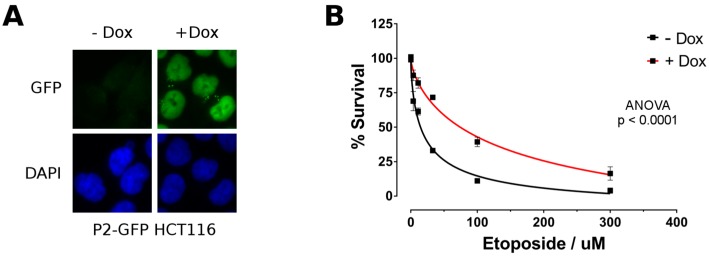
P2-GFP expression increases the resistance of HCT116 colorectal cancer cells to etoposide. The inducible HCT116 P2-GFP cell line was incubated 48 h with or without doxycycline to induce or not the expression of the P2-GFP construct. (**A**) Induction of P2-GFP expression was validated by monitoring GFP fluorescence by microscopy. Nuclei were counterstained using DAPI; (**B**) HCT116 P2-GFP cells induced (+Dox) or not (−Dox) with doxycycline were treated with increasing concentrations of etoposide for 72 h and cell viability was measured by alamarBlue. Etoposide sensitivity was determined by plotting the percentage of cell survival to etoposide concentration and fitting a four-parameter dose-response curve. *n* = 3; *p*-value calculated by the two-way ANOVA test.

To further evaluate whether HNF4α played a functional role during the DNA damage response, we generated a stable HCT116 cell line for P2-GFP using the Flp-In T-REx system allowing genome integration at a single and known location under the control of a doxycycline-inducible CMV promoter. Immunofluorescence microscopy performed on cells incubated or not with doxycycline confirmed induced nuclear expression of P2-GFP under these conditions (Figure 7A). HCT116 cells negative (−Dox) or positive (+Dox) for P2-GFP expression were next exposed to different concentrations of etoposide during 72 h. Cell viability for P2-GFP postive cells was significantly enhanced for each etoposide concentrations tested (compare red curve with black curve, Figure 7B). This observation supported that HNF4α can protect colorectal cancer cells during the DNA damage response.

## 3. Discussion

HNF4α expression has been shown to be crucial for the survival of CRC cell lines in culture and during intestinal polyposis in mice [11,12,37]. Recent work supported that P2 isoform classes of HNF4α promote cancer cell survival [14]. To further investigate the role of HNF4α in carcinogenesis, two separate approaches were used to identify proteins that interact with the HNF4α7 isoform as a representative of P2 promoter-driven HNF4α isoforms in transformed and colorectal cancer cell lines. The differences in approach between the GFP-Trap and BioID served a twofold purpose. Firstly, the two separate techniques created redundancy in the experimental approach. This redundancy permitted for a more robust identification of proteins was found to associate with P2-HNF4α. The results from both techniques were compared to each other qualitatively to demonstrate whether any targets were identified in both conditions. Secondly, the two techniques varied in the mechanism of detection. GFP-Trap is dependent on a physical association between proteins [38] that can withstand cell lysis. BioID is dependent on the physical proximity between the active site of the BirA enzyme moiety of the fusion protein construct without regard for maintaining protein-protein interactions post-lysis [32,38]. It is thus expected for the BioID approach to be more efficient in identifying interacting partners with lower affinity, but we chose to further limit our analysis to common targets identified from both techniques as a more stringent approach. This strategy turned out to be successful given that we were able to validate endogenous interactions of P2-HNF4α with PARP1, RAD50 and DNA-PKcs in colorectal cancer cell lines. 

DNA repair is not a role traditionally ascribed to HNF4α or more generally speaking, to other tissue-specific master gene regulators. Recent findings have however identified specific transcription factors to act as DNA repair entities independently of their classical transcriptional role when recruited at DNA damage sites [23]. One of the most convincing studies to this end was recently provided with the nuclear orphan receptor NR4A involved in neurons and hematopoietic lineage differentiation [39]. NR4A was shown to interact with DNA-PKcs and to translocate to DNA double-strand breaks upon the DNA damage exposure. Interestingly, PARP-1 was shown to be functionally involved for the translocation of NR4A to DNA damage sites during this process [39]. Given that HNF4α belongs to the nuclear receptor superfamily and was shown here to interact with similar DNA repair entities, it is tempting to speculate that P2-HNF4α could mechanistically harbor similar features in this context. Our data support that P2-HNF4α interaction with the DNA repair protein machinery does not require the DNA binding motif nor the transactivating AF-2 domain, both being crucial for regulating transcription. Whether P2-HNF4α interactions with these proteins are relevant for chromatin-modifying activities during the DNA repair processes will have to be further investigated. 

Our analysis showed that P2-HNF4α interacts with PARP1, a crucial component of various pathways for the DNA repair [36]. In addition, our quantitative proteomics approaches identified several other DNA repair components predicted to interact with P2-HNF4α providing then alternative means to affect the DNA damage response. In support of this, nucleophosmin (NPM1) and p53, both identified in the GFP-Trap procedure (Supplementary Table S1), are known to functionally associate [40]. The protein p53 roles as a universal tumor suppressor gene as well as guardian of genome integrity is exerted by numerous cellular mechanisms [22]. Moreover, p53 was shown to physically interact with the ligand-binding domain of HNF4α [41], an interaction that we have confirmed on our own (data not shown). NPM1 is a multifunctional protein involved in the maintenance of genome integrity and was shown to regulate the base excision repair (BER) pathway [42]. In addition, MSH2 and MSH6 proteins were identified as interactors of P2-HNF4α with the BioID technique (Supplementary Tables S2 and S3). Both proteins are important in first identifying DNA mismatches after replication and to then stimulate DNA repair [43]. These observations indicate that P2-HNF4α might interfere with several distinct DNA repair pathways in the context of CRC cells. 

## 4. Material and Methods

### 4.1. Cell Culture and SILAC Labelling 

The HEK293T human fetal kidney transformed cell line and colorectal cancer cell lines HCT116, HT-29 and LoVo were obtained from the American Type Culture Collection (ATCC, Manassas, VA, USA). HEK293T cells were cultured in the DMEM medium, HCT116 cells in McCoy’s medium while LoVo cells in the F-12K medium. All culture media were supplemented with 10% fetal bovine serum (Wisent Inc., St-Bruno, QC, Canada), 10 mM HEPES, and 1× GlutaMAX (Gibco, Gaithersburg, MD, USA). Cells were maintained at 37 °C in a humidified atmosphere containing 5% CO_2_. For the SILAC culture, cells were incubated in Light or Heavy media for at least four passages at a sub-cultivation ratio of 1:3 to 1:5 in order to fully incorporate isotopically labeled amino acids into their proteomes. Light media was composed of L-Arginine R0 and L-Lysine K0 (Sigma-Aldrich, Oakville, ON, Canada) and Heavy media of L-Arginine R10 and L-Lysine K8 (Cambridge Isotope Laboratories Inc., Andover, MA, USA). SILAC media were composed of DMEM (no arginine, no lysine, no glutamine; Gibco) containing supplements with 75 µg/mL of L-Arginine, 113 µg/mL of L-Lysine, 10% triple dialyzed fetal bovine serum (Gibco), 1× GlutaMAX (Gibco,), 10 mM HEPES and 100 U/mL penicillin/streptomycin.

### 4.2. Plasmid Construction and Generation of Stable Cell Lines

The P2-GFP recombinant lentiviral expression vector was synthesized and subcloned into the pLenti6/V5 vector (Invitrogen-Thermo Fisher Scientific, Waltham, MA, USA) by Feldan Inc. (Quebec, QC, Canada). Briefly, an expression-optimized DNA sequence based on the mRNA of HNF4α7 isoforms (NM_001030003.1) and fused at its 3′ end to the eGFP sequence (AF188479) with the linker sequence TTCACTAGTTCAGAATTC was synthesized. This DNA fragment was inserted between the *BamHI* and *EcoRV* sites of the in-house modified pLenti6/V5 vector with the addition of a Kozak sequence. Mutated P2-GFP constructs were generated through the PCR site-directed mutagenesis using primer pairs (Supplementary Table S4). Briefly, the P-box sequence located in the first zinc finger of HNF4α (amino acids 55 to 60) was either removed or replaced by the glucocorticoid receptor P-box sequence (CGSCKV) for the ΔP-box and GR P-box mutants, respectively. The ΔA/T-box and the ΔAF-2 mutant were generated by deleting the A-box and T-box region (amino acids 113 to 129) or the cofactor interacting region AF-2 (amino acids 347 to 352), respectively. The C93R mutant was generated by the substitution of a cysteine for an arginine at the position 93 in the second zinc finger of HNF4α. The P2-HNF4α-BirA recombinant lentiviral expression vector was generated by the PCR-amplification of the HNF4α7 sequence template and subcloned into the *BamHI* and *XhoI* restriction sites of the modified pLenti6/V5 vector to remove the 3′ GFP tag. The sequence of BirA-myc-tag (birA mutant R118G with a Myc-tag at the C-terminal end) was then amplified by PCR from the pGLAP5-BirA vector [44] and inserted in frame at the 3′ end of the HNF4α7 sequence between the *SpeI* and *SacII* restriction sites. Stable cell lines were generated by infection with lentivirus particles and selection with the use of 8 µg/mL of blasticidin. To generate a doxycycline inducible P2-GFP HCT116 cell line, the HNF4α7 sequence was amplified by PCR, inserted into the pENTR11 vector and then further transferred into the pgLAP5.2-GFP vector [45] by Gateway cloning, via an LR reaction according to the manufacturer’s instructions (Thermo Fisher Scientific, Waltham, MA, USA). Transfections to generate the stable line were performed with HCT116 Flp-In T-Rex cells, when these had reached approximately 70% confluence in a 60 mm petri dish using Lipofectamine LTX (Thermo Fisher Scientific) as a transfection agent. A quantity of 500 ng of plasmid DNA was mixed with 4.5 μg of the Flp-Recombinase expression vector pOG44 (Thermo Fisher Scientific), 5 μL of Plus Reagent (Thermo Fisher Scientific) and Opti-MEM medium (Thermo Fisher Scientific) to complete the reaction at a volume of 300 μL. A second mixture was carried out simultaneously between 8 μL of lipofectamine LTX and 292 μL of Opti MEM medium. These two reactions were incubated for 5 min at room temperature before being mixed and incubated for 30 min at room temperature. Meanwhile, HCT116 cells were washed once with 1× PBS followed by the addition of 2 mL of DMEM culture medium (Thermo Fisher Scientific) containing 10% FBS (Wisent, St. John the Baptist, QC, Canada). The selection was started 24 h later, adding the antibiotics blasticidine S (5 μg/mL) and hygromycin B (100 μg/mL). The clonal population was maintained over time by the continued use of these antibiotics, and the induction of the P2-GFP construct was achieved via the addition of 2.5 μg/mL doxycycline (Clontech Laboratories, Mountain View, CA, USA) in the medium at the desired moment.

### 4.3. Quantitative PCR

Total RNA from cells was extracted using the QIAGEN RNeasy kit and subjected to DNAse treatment (Roche, Laval, QC, Canada). RNA of 2 µg quantified by NanoDrop 2000 (Thermo Scientific) were used for reverse transcription with AMV-RT (Roche) and synthesized cDNAs diluted to 5 ng/µL prior to the qPCR analysis. qPCR reactions were done using a Lightcycler 96 apparatus (Roche) in a total volume of 20 µL containing 10 µL of FastStart Essential DNA Green Master (Roche), 500 mM of each primer and 10 ng of cDNAs (Supplementary Table S4). Amplicons were designed to range between 150 and 200 nucleotides and analyzed by melting curves after each run to discriminate for non-specific amplifications. All qPCR reactions were run with a no-DNA control to confirm the absence of DNA contamination. The relative expression of each target genes was calculated using the formula E_T_^CqT(calib)-CqT(sampl)^ × E_R_^CqR(sampl)-CqR(calib)^ where E_T_/E_R_ and C_q_T/C_q_R are the reaction efficiency (E) for quantification cycle (C_q_) of the target (T) and reference (R) gene for both the experimental sample (sampl) and the calibrator sample (calib).

### 4.4. Immunoprecipitations and Immunoblots

Total protein extracts from LoVo and HT-29 cells were prepared using the Triton buffer (Triton X-100 1%, 150 mM NaCl, 20 mM Tris-HCl, pH 7.5) followed by three rounds of 10 s sonication at 20% (Fisher FB-120 Sonic Dismembrator) to completely lyse nuclei. Immunoprecipitations were performed with an overnight incubation of 400 µg of protein extracts and 4 µg of anti-HNF4α (Santa Cruz Biotechnology, Dallas, TX, USA; sc-6556) or anti-DNA-PKcs (Invitrogen-Thermo Fisher Scientific; MA5-13404) followed by a two-hour incubation of 20 µL of MagnaChIP Protein G beads (Millipore, Billerica, NA, USA). Co-immunoprecipitated proteins were then eluted from the isolated beads by a 10 min incubation at 95 °C in NuPAGE LDS Sample Buffer 1× (Invitrogen-Thermo Fisher Scientific). Immunoblots were done using the NuPAGE Bis-Tris 4–12% gel and the XCell SureLock system (Invitrogen-Thermo Fisher Scientific) using 2 µg or 40 µg of total protein extracts or 50% to 100% of the immunoprecipitated samples. Anti-GFP (Santa Cruz Biotechnology), anti-PARP-1 (Cell Signaling, Danvers, MA, USA; 9542), anti-DNA-PKcs (Invitrogen-Thermo Fisher Scientific; MA5-13404), anti-RAD50 (Abcam, Cambridge, MA, USA; ab89), anti-βactin (Millipore, Billerica, USA; MAB1501R) and several anti-HNF4α (R&D Systems, Minneapolis, MN, USA; H1415) (Santa Cruz Biotechnology; sc-6556 and sc-374229) were used for immunoblotting. For the immunoprecipitations of HNF4α mutant constructs, 3.5 × 10^6^ of HCT116 cells were seeded in 60 mm dishes and transfected with 14 µg of pDEST47-HNF4α mutant vectors using Lipofectamine 2000 (Thermo Fisher Scientific) before total protein was extracted 48 h later as described above. Protein extracts of 400 µg were then incubated overnight with 20 µL of GFP-Trap beads (Chromotek, Hauppauge, NY, USA) or control protein-A sepharose CL-4B beads (GE Healthcare, Mississauga, ON, Canada) before being washed six times in the Triton buffer and eluted 10 min at 95 °C in 20 µL NuPAGE LDS Sample Buffer 1×. Immunoblots were performed using 2 µg or 40 µg of total protein extracts or 20% to 50% of the immunoprecipitated samples and immunoprecipitation validated with an anti-GFP (Roche; 1181446000).

### 4.5. GFP-Trap Assay and In-Gel Digestion

HEK293T cells were infected with P2-HNF4α-eGFP or empty control lentiviruses and selected with 8 µg/mL of blasticidin for 5 to 10 days until all cells in an uninfected control Petri dish were killed by the selection. In order to perform quantitative mass spectrometry, the proteome of P2-HNF4α-eGFP and empty control cells were labelled using the SILAC technique by successive amplification in heavy and light media, respectively. To perform the GFP-TRAP assay, two Petri dishes of 150 mm were used as starting material for each condition. Cells were recovered using trypsin and washed twice carefully with D-PBS 1×. Nuclear protein extracts were prepared as previously described [46]. Briefly, cells were resuspended in a hypotonic buffer (10 mM HEPES pH 7.9, 1.5 mM MgCl_2_, 10 mM KCl, 0.5 mM DTT) and dounced-homogenized 25 times with a tight pestle to isolate nuclei. Nuclei were recovered by centrifugation and further purified on a sucrose gradient. Clean nuclei pellets were resuspended in RIPA 1× buffer (50 mM Tris pH 7.5, 150 mM NaCl, 1% Nonidet P40 substitute, 0.5% Sodium deoxycholate) and sonicated five times for 10 s at 25% on a Fisher FB-120 Sonic Dismembrator with a 1/8-inch probe (Thermo Fisher Scientific). Nuclear extracts were further diluted in RIPA 1× buffer and incubated one hour at 4 °C with 40 µl of GFP-TRAP beads (Chromotek, Hauppauge, NY, USA) to immunoprecipitate the P2-HNF4α-eGFP protein and interacting partners. Beads were washed three times with RIPA 1× and two times with PBS 1× buffer. After the last wash, beads from the different conditions were pooled together before recovering the proteins by heating the beads at 95 °C for 10 min in NuPAGE LDS Sample Buffer 1× (Invitrogen-Thermo Fisher Scientific). At all steps, a protease inhibitor cocktail (Sigma-Aldrich, Oakville, Canada) was added to avoid protein degradation. The recovered proteins were then analyzed by mass spectrometry after being reduced to peptides by in-gel digestion as previously described [47]. 

### 4.6. BioID Assay

HEK293T and HCT116 cells were infected with lentiviruses expressing the P2-HNF4α-BirA construct or a control empty vector. Cells were selected and amplified in the SILAC media as described above. To induce in vivo biotinylation of potential protein partners, cells were incubated 24 h in media containing 50 µM of biotin (Sigma-Aldrich, Oakville, ON, Canada) before being collected by dissociation with trypsin and washed twice in D-PBS 1×. Nuclear extracts were prepared as described above and diluted in RIPA 1× buffer. To immunoprecipitate the biotinylated proteins, 50 µL of streptavidin coupled beads (GE Healthcare, Mississauga, ON, Canada) was added to the nuclear extract and incubated 2 h at 4 °C. Beads were then washed three times with RIPA 1× and five times with 20 mM ammonium bicarbonate to remove unspecific interactions. After the last wash, the beads from the different conditions were combined and proteins directly converted to peptides using the on-Beads digestion technique described previously [44].

### 4.7. Etoposide Sensitivity Assay

P2-GFP HCT116 inducible cells were seeded in 48-well plates (Corning, Durham, NC, USA) at a density of 15,000 cells per well. The following day, cells were cultivated 48 h in the presence of 2.5 µg/mL of doxycycline (Biobasic, Markham, ON, Canada) to induce the P2-GFP expression, or in the presence of an equivalent amount of water to serve as controls. Media were changed and cells were incubated 72 h in the presence of 3-fold dilution series of etoposide (Selleckchem, Houston, TX, USA) ranging from 3.7 µM to 300 µM in the presence or not of doxycycline. Cell viability was determined with the alamarBlue Cell Viability Reagent following the manufacturer’s instructions (Invitrogen-Thermo Fisher Scientific). Fluorescence reading was achieved with the FlexStation 3 device (Molecular Devices, San Jose, CA, USA) and cell viability was normalized to untreated control cells. Curve fitting was done with a four-parameter dose-response curve on the GraphPad Prism version 7.00 (GraphPad Software, La Jolla, CA, USA) and two-way ANOVA was performed for statistical analysis.

### 4.8. Fluorescence Microscopy

P2-GFP HCT116 inducible cells were seeded on a round glass coverslip (Thermo Fisher Scientific) in a 24-well plate to cover approximately 20% of the surface and induced 48 h with 2.5 µg/mL of doxycycline or not. Cells were fixed in 4% formaldehyde for 30 min and permeabilized 5 min in PBS Triton X-100 0.1%. Samples were blocked in PBS BSA 2% for 30 min and nuclei stained for 10 min in 0.3 µg/mL of DAPI. Coverslips were mounted on glass slides with Thermo Scientific Shandon Immu-Mount (Thermo Fisher Scientific) and cells were observed on a Zeiss Axiovert 200M fluorescent microscope (Carl Zeiss, Toronto, ON, Canada). 

### 4.9. Mass Spectrometry Analysis

Trypsin digested peptides were loaded and separated onto a nanoHPLC system (Dionex Ultimate 3000). 10 µL of the sample (2 µg) was first loaded with a constant flow of 4 µL/min onto a trap column (Acclaim PepMap100 C18 column, 0.3 mm id × 5 mm, Dionex Corporation, Sunnyvale, CA, USA). Peptides were then eluted off towards an analytical column heated to 40 °C (PepMap C18 nano column (75 µm × 50 cm)) with a linear gradient of 5–35% of solvent B (90% acetonitrile with 0.1% formic acid) over a 4 h gradient at a constant flow (200 nL/min). Peptides were then analysed by an OrbiTrap QExactive mass spectrometer (Thermo Fischer Scientific) using an EasySpray source at a voltage of 2.0 kV. Acquisition of the full scan MS survey spectra (m/z 350–1600) in profile mode was performed in the Orbitrap at a resolution of 70,000 using 1,000,000 ions. Peptides selected for fragmentation by collision-induced dissociation were based on the ten highest intensities for the peptide ions from the MS survey scan. The collision energy was set at 35% and resolution for the MS/MS was set at 17,500 for 50,000 ions with maximum filling times of 250 ms for the full scans and 60 ms for the MS/MS scans. All unassigned charge states as well as singly, seven and eight charged species for the precursor ions were rejected, and a dynamic exclusion list was set to 500 entries with a retention time of 40 s (10 ppm mass window). To improve the mass accuracy of survey scans, the lock mass option was enabled. Data acquisition was done using the Xcalibur version 2.2 SP1.48 (Thermo Fischer Scientific). Identification and quantification of proteins identified by mass spectrometry was done using the MaxQuant software version 1.5.2.8 [48]. Biological replicates were done twice and combined together for the MaxQuant analysis. Quantification was done with light (Lys 0 and Arg 0) and heavy (Lys 8 and Arg 10) labels and considering a trypsin digestion of the peptides with no cleavages on lysine or arginine before a proline. A maximum of two missed cleavages were allowed with methionine oxidation and protein N-terminal acetylation as variable modifications of proteins and carbamidimethylation as fixed modification. The maximum number of modifications allowed per peptide was set to five. Mass tolerance was set to a maximum of 7 ppm for the precursor ions and 20 ppm for the fragment ions. The minimum length of peptides to be considered for quantification was set to seven amino acids and the false discovery rate threshold set to 5%. The minimum number of peptides to be used for the identification of proteins was set to one but only proteins identified with two or more peptides were considered in further analysis. Protein quantification was calculated using both unique and razor peptides. To remove the common background contaminants from the protein partners identified with the BioID assay, the proteins identified by MaxQuant software were analyzed by the Crapome database version 1.1 [49] to score interactions. Briefly, the spectral counts of the proteins identified in both duplicates of the BirA assay for the P2-HNF4α-BirA condition were compared to the empty control condition as well as twelve (CC532, CC533, CC534, CC535, CC536, CC537, CC538, CC553, CC554, CC555, CC556 and CC557 controls) HEK293 BirA-Flag negative control pulldowns available from the CRAPome database for the HEK293T BioID assay or sixteen (CC539, CC540, CC541, CC542, CC543, CC544, CC545, CC546, CC547, CC548, CC549, CC550, CC551, CC552, CC558 and CC559) HeLa BirA-Flag negative control pulldowns for the HCT116 BioID assay. The scoring interaction for each protein was determined based on the fold change results (FC_B; default setting used) computed by averaging the three highest normalized spectral counts across all controls (our empty control and the CRAPome database selected controls). Interactions with a fold change of 2.0 or higher were considered as real targets from the BioID assays and were the only ones considered for further analysis. Biological functions associated with the identified HNF4α proteins partners in the GFP-TRAP or BioID assay were determined using the Functional Annotation Chart tool of the DAVID database version 6.8 [31]. Analyses were done based on the whole human genome background file of DAVID’s database. Functional protein interaction networks were analyzed using STRING v10.5 [50] based on a minimum required interaction score of 0.400. The protein database used for protein identification was the *Homo sapiens* proteome (88 354 entries) downloaded from the Uniprot database on the 07/16/2013. The mass spectrometry data have been deposited to the ProteomeXchange Consortium via the PRIDE partner repository with the dataset identifier PXD008365. 

## 5. Conclusions

In conclusion, our report identified interaction networks between HNF4α and multiple proteins characterized with a role in DNA repair. The precise mechanisms by which HNF4α acts during DNA repair biological processes must be complex and opens on a novel field of investigation for this central regulator involved in differentiation of endodermal-derived epithelial tissues as well as during the control of cell growth in tumorigenesis.

## Supplementary Materials

The following are available online at https://www.mdpi.com/2072-6694/11/5/626/s1, Figure S1: P2-GFP construct modulates HNF4α’s target genes expression in HCT116 cells, Figure S2: P2-BirA induces endogenous expression of VIL1 and HNF1A in HEK293T cells, Table S1: Proteins enriched more than 1.5 times in P2-GFP pull-downs (293T), Table S2: Proteins enriched more than two times in P2-BirA BioID assay (293T), Table S3: Proteins enriched more than two times in P2-BirA BioID assay (HCT116), Table S4: Primer pairs used for qPCR and PCR site-directed mutagenesis of the P2-GFP construct.
